# Identification of p53 and Its Isoforms in Human Breast Carcinoma Cells

**DOI:** 10.1155/2014/618698

**Published:** 2014-01-05

**Authors:** Zorka Milićević, Vladan Bajić, Lada Živković, Jelena Kasapović, Uroš Andjelković, Biljana Spremo-Potparević

**Affiliations:** ^1^Laboratory of Molecular Biology and Endocrinology, “Vinča” Institute of Nuclear Sciences, University of Belgrade, Mihaila Petrovića Alasa 12-14, P.O. Box 522, 11 001 Belgrade, Serbia; ^2^Biomedical Department, Institute for Pharmaceutical Research and Development, Galenika a.d., Pasterova 2, 11 000 Belgrade, Serbia; ^3^Department of Biology and Human Genetics, Institute of Physiology, Faculty of Pharmacy, University of Belgrade, Vojvode Stepe 450, 11 000 Belgrade, Serbia; ^4^Department of Chemistry, Institute for Chemistry, Technology and Metallurgy, University of Belgrade, Studentski trg 12-16, 11000 Belgrade, Serbia

## Abstract

In breast carcinoma, disruption of the p53 pathway is one of the most common genetic alterations. The observation that the p53 can express multiple protein isoforms adds a novel level of complexity to the outcome of p53 mutations. p53 expression was analysed by Western immunoblotting and immunohistochemistry using monoclonal antibodies DO-7, Pab240, and polyclonal antiserum CM-1. The more frequently p53-positive nuclear staining has been found in the invasive breast tumors. One of the most intriguing findings is that mutant p53 appears as discrete dot-shaped regions within the nucleus of breast cancer cells. In many malignant cells, the nucleolar sequestration of p53 is evident. These observations support the view that the nucleolus is involved directly in the mediation of p53 function or indirectly by the control of the localization of p53 interplayers. p53 expressed in the nuclear fraction of breast cancer cells revealed a wide spectrum of isoforms. p53 isoforms ΔNp53 (47 kDa) and Δ133p53**β** (35 kDa), known as dominant-negative repressors of p53 function, were detected as the most predominant variants in nuclei of invasive breast carcinoma cells. The isoforms expressed also varied between individual tumors, indicating potential roles of these p53 variants in human breast cancer.

## 1. Introduction

The tumor suppressor p53 plays a vital role in the response to DNA damage and has been classified as a “guardian of the genome” due to its ability to coordinate multiple and diverse signaling pathways involved in this response [1]. Gene expression microarrays have revealed that p53-regulated genes are not limited to those involved in cell cycle arrest and apoptosis. Many other gene clusters associated with diverse processes such as DNA repair, transcription, cell adhesion, cell mobility, metabolism, and membrane functions are also affected by p53 activity. The complex repertoire of p53-regulated genes further highlights the imperative need to understand how p53 selects its key target genes. Mutation of p53 is a common occurrence in many cancers and is associated with tumor progression, resistance to chemotherapy, and poor prognosis [2]. A study of breast cancers found that p53 mutation frequency was not related to nodal involvement or tumor size [3], although another study found a marginally increased frequency in recurrent tumors [4]. It was also reported that inactivation of p53 may be due to inhibition of the function of wild-type p53 itself [5]. In addition, breast cancer patients have been found to have tumors which are characterized by changes in the levels of wild-type p53 transcripts [6]. This affects the levels of downstream products and understanding the role of p53 in tumorigenesis would perhaps require the characterization of mutations in proteins that physically partner p53 and may control its levels. p53 gene family members express multiple mRNA variants due to multiple splicing and alternative promoters. Hence, p53 gene family members express different forms of p53 protein containing different domain of the protein (isoforms). This indicates that wild-type p53 activity may be modulated in the presence of p53 isoforms. The traditional belief has been that each p53 protein isoform may have specific biological activities independent of full-length p53 [7–9]. While many developments have been made in understanding the biology of p53, this has been accompanied by an increased perception of its complexity [10]. In the network of cancer-related genes, pathways are the frame by which we can understand the network logically. The goal of the molecular analysis of human cancer is to know all genetic changes in a cancer cell, the order in which they appear, and what the products of these genes do. How many different possibilities there are at the molecular level for a mammary tumor to arise remains to be established. In the present study, marker p53 is selected based on the most frequently mutated gene, and its expression level in breast cancer specimens was detected by immunohistochemistry (IHC), which is generally used for regular pathological detection. Although not every study of p53 expression contains unequivocal information about the pattern of expression, from the data available to date, it would be reasonable to suggest that this would be highly relevant in the prediction of the course of the disease. We also compared the pattern of expression of p53 isoforms which appear to be involved in malignant transformation and tumor progression. This attempt is an extension of our continued interest in investigating the mode of action of p53 family members and its isoforms.

## 2. Materials and Methods

### 2.1. Reagents

All reagents were purchased from Sigma (St. Louis, MO, USA) and Merck (Darmstadt, Germany). Rabbit anti-human p53 polyclonal antibody (CM-1) was obtained from Midgley et al. [11]. Biotinylated swine anti-rabbit Ig serum (E-353), mouse anti-human p53 monoclonal antibody (clone DO-7, M-7001), biotinylated rabbit anti-mouse antibody (E-354), and ABComplex-HRP (K-377) were purchased from Dako Denmark (Glostrup, Denmark). Monoclonal antibody Pab240 (sc-99), HRP-conjugated rabbit anti-mouse IgG (sc-358917), and rabbit anti-actin antibody (C-11: 1615) were purchased from Santa Cruz Biotechnology (Santa Cruz, CA, USA). HRP-conjugated goat anti-rabbit IgG (SAB-301) were purchased from Millipore (Billerica, MA 01821, USA). Rabbit anti-actin antibody (C-11: 1615) was obtained from Santa Cruz Biotechnology (Santa Cruz, CA, USA). HRP-conjugated goat anti-rabbit IgG (SAB-301) was purchased from Millipore (Billerica, MA 01821, USA). Human breast carcinoma cell line (CL-239 M) was purchased from BioGenex (Fremont, CA, USA).

### 2.2. Subjects and Breast Cancer Samples

The study included 47 breast cancer patients (age in years: mean = 57, min. = 32, and max. = 86) from the Institute of Oncology and Radiology of Serbia, Belgrade, Serbia. All patients were newly diagnosed and tumors were clinically categorized as stages II and III, according to the classification of the International Union Against Cancer [12]. Primary breast cancer cases were classified according to TNM classification and tumors were histologically diagnosed as ductal (30), lobular (12), tubular (3), and medullary (2) type. Ten normal breast tissues were selected from macroscopically normal areas and 6 from nontumor patients. During the study period, patients were not submitted to endocrine, chemotherapy, or radiotherapy. The patients and the controls were nonsmokers and they used no alcohol consumption, hormones, oral contraceptives, or dietary supplements with antioxidants. None of the subjects had concomitant diseases such as diabetes mellitus, rheumatoid arthritis, liver disorders, or any other malignancies. According to the ethical guidelines of the Helsinki Declaration, informed consent was obtained from all participants and the protocol used in this study was approved by the Ethics Committee of Institute of Oncology and Radiology of Serbia, University of Belgrade, Belgrade, Serbia.

### 2.3. Tissue and Cellular Fractionation

The normal and cancer tissues were surgically dissected and frozen. The nuclear fractions of normal and tumor tissues were obtained by differential centrifugation [13]. Briefly, the samples of normal and neoplastic tissues were minced by fine dissection and homogenized in 0.25 M sucrose, 5 mM MgCl_2_, 0.5% Triton X-100, 1 mM phenylmethylsulfonylfluoride (PMSF), and 50 mM TRIS-HCl, pH 7.4, followed by centrifugation of the homogenate at 800 xg for 10 min (Sorvall RC-5B, Sorvall Ltd., Stevenage, England). The supernatants were spun at 11000 xg at 4°C for 25 min and the resulting supernatant was again centrifuged at 20000 xg at 4°C for 45 min (Beckman L7-55 centrifuge, Beckman Instruments Inc., Palo Alto, CA, USA). The cytosolic fraction was obtained by further centrifugation of supernatant at 100000 xg at 4°C for 60 min. The pellet obtained after first centrifugation was homogenized in ice-cold buffer (2.2 M sucrose, 5 mM MgCl_2_, 0.5% Triton X-100, 1 mM PMSF, and 50 mM Tris-HCl, pH 7.4) and further purified by centrifugation. After this step the pellets representing the soluble nuclear proteins were resuspended in 0.25 M sucrose, 5 mM MgCl_2_, 0.5% Triton X-100, 1 mM PMSF, and 50 mM Tris-HCl, pH 7.4. The protein concentration was determined by Lowery et al. method [14], and samples were aliquoted and stored at −20°C.

### 2.4. SDS-Polyacrylamide Gel Electrophoresis and Immunoblot Analysis

Proteins isolated from normal and breast cancer cells were suspended in sample loading buffer (0.1 M Tris, pH 6.8, 20% glycerol, 4% SDS, 0.04% bromphenol blue, 10% *β*-mercaptoethanol) and separated electrophoretically on 12% acrylamide gels according to procedure of Laemmli [15]. Equal amounts of nuclear proteins (50 *μ*g) per line were loaded and run on 12% SDS-PAGE (Mini-protean 3 Cell, Bio-Rad Laboratories, Inc., Hercules, CA, USA). Separated proteins were transferred onto nitrocellulose membrane (Amersham Hybond, GE Healthcare Bio-Sciences AB, Uppsala, Sweden) according to the procedure of Towbin et al. [16]. Transfer of proteins from the acrylamide gel to nitrocellulose was performed in transfer buffer (25 mM Tris, 192 mM glycine, 0.1% SDS, 20% methanol, pH 7.5) at 30 V, 40 mA overnight at 4°C (Trans-Blot SD Electrophoretic Transfer Cell, Bio-Rad, Hercules, CA, USA). Nitrocellulose filters were blocked overnight with blocking buffer (1% BSA in 50 mM Tris, 0.9% NaCl, 0.05% Tween 20, pH 7.5). The filters were then incubated for 1 h 45 min in buffer (50 mM Tris, 0.9% NaCl, 0.05% Tween 20, pH 7.5) plus primary antibody CM-1 at the optimal dilution 1 : 1000. The CM-1 antibody is a rabbit high-titre polyclonal antiserum raised against human recombinant wild-type p53 protein [11]. After 1 h of incubation with the biotinylated swine anti-rabbit Ig serum (diluted 1 : 1000) and strept ABComplex-HRP conjugated (diluted 1 : 100) the proteins bands were visualized in a HRP substrate diaminobenzidine tetrahydrochloride (DAB). The same membranes were stripped and probed for *β*-actin (dilution 1 : 10000) as an internal loading control. To detect actin with rabbit polyclonal antibody, we used the HRP-conjugated goat anti-rabbit (1 : 2000) antibodies as secondary antibodies. Reproducibility was assessed by repeating the protein extraction and the SDS-PAGE three times. The protein level of each band was quantified by transmission scanning and analyzed by Gel-Pro Analyzer 3.1.

### 2.5. Immunohistochemical Analysis

Immunohistochemical (IHC) staining was performed on 3 *μ*m tissue sections by an avidin-biotin-peroxidase complex (ABC) technique [17] using both frozen and formalin-fixed paraffin-embedded tissues. For formalin-fixed, paraffin-embedded sections, we applied the microwave oven heating technique, which showed to be effective for the retrieval of masked epitopes of many antigens. The primary antibodies used were mouse monoclonal antibodies (clone DO-7) recognizing a denaturation-resistant epitope between amino acids 35 and 45 and have been shown to recognize both the wild-type and mutant forms of p53 protein [18]. Briefly, after dewaxing, endogenous peroxidase activity was blocked by incubating the sections with methanol containing 3% hydrogen peroxide for 20 min. After preincubation with normal rabbit serum (diluted 1 : 5) for 20 min, a three-step immunoperoxidase procedure was applied: first, with the primary antibodies DO-7 at the appropriate working dilution 1 : 50 in 0.1% BSA overnight at 4°C, second, with biotinylated rabbit anti-mouse immunoglobulin (diluted 1 : 250 in 0.1% BSA) for one hour at room temperature, and third, with strept ABComplex-HRP (diluted 1 : 100) for 40 min. Finally, the sections were visualized by incubation with diaminobenzidine tetrahydrochloride (DAB) in hydrogen peroxide substrate with 50 mg of imidazole for 10 min. The sections were counterstained with methyl green (0.5%, 2 min) or Harris haematoxylin to visualize nuclei. We also used the alkaline phosphatase anti-alkaline phosphatase (APAAP) immunohistochemical technique [19]. Negative control slides were processed with each slide run and excluded the primary antibody but included all other steps of the procedure. A human breast carcinoma cell line, which expresses p53 suppressor gene product, is used as the positive control. The IHC staining was semiquantitatively scored by 2 of the authors. We studied the tissue samples by assessing the site of staining, the proportion of cell staining (counting at least 1000 cells/sample), and the intensity of staining: weak (1+), moderate (2+), or strong (3+). Scoring was as follows: 1%–10% tumor cell nuclei staining for p53 = 1+; 11%–30% tumor cell nuclei staining for p53 = 2+; 31%–50% tumor cell nuclei staining for p53 = 3+; >50% tumor cell nuclei staining for p53 = 4+.

### 2.6. Statistical Analysis

The significance of differences between different experimental values was assessed by means of ANOVA and Student's *t*-test. Data were tested at a statistical significance level of *P* < 0.05 and expressed as mean ± SEM. All statistical manipulations were performed using the SPSS for Windows software system.

## 3. Results 

### 3.1. Expression of p53 Protein in Breast Carcinoma: Immunohistochemistry and Western Blot Analysis

**Figure 1:**
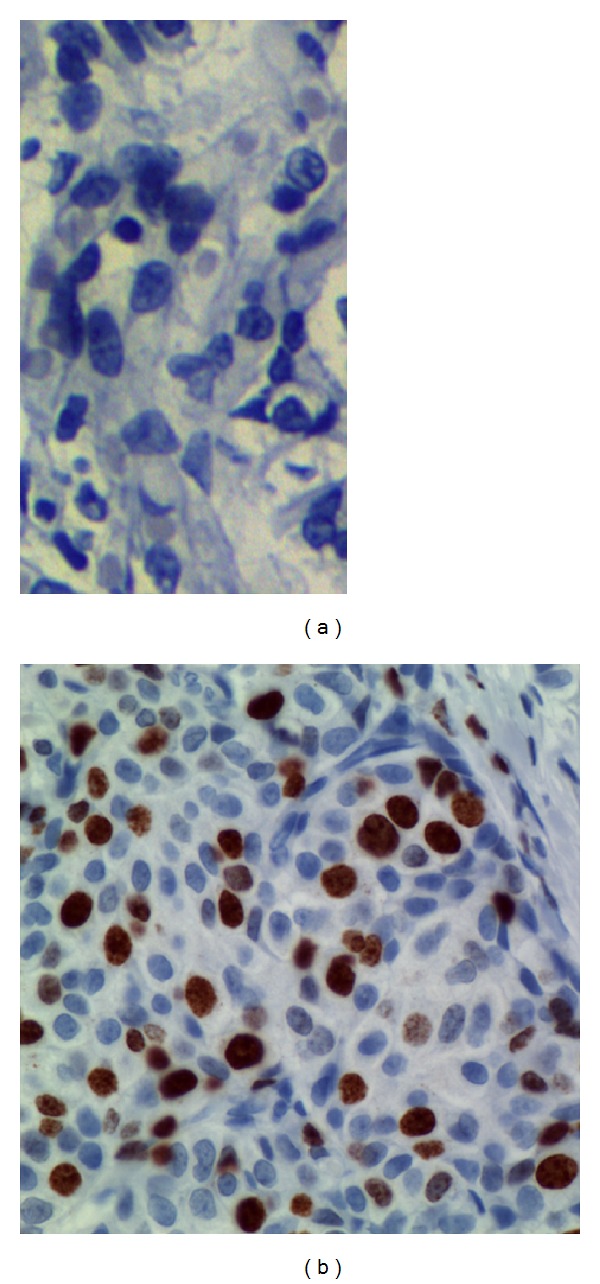
p53 immunohistochemical staining in normal breast tissue and breast cancer. (a) No p53 immunoreactivity was observed in the normal breast cells. (b) A mutant case with strongly positive nuclear staining. Positive p53 immunoreaction in the tumor area is indicated by brown color. Negative nuclei show only the counterstain, indicated by the blue color. Immunostaining was performed with the mouse anti-human p53 monoclonal antibody DO-7. (Immunoperoxidase staining with nuclear counterstain with hematoxylin. Magnification: ×63.)

**Figure 2:**
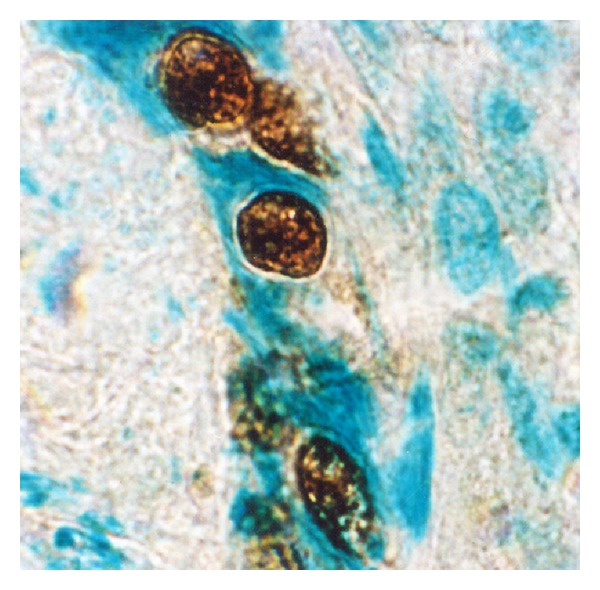
p53 immunostaining in malignant cells from an invasive (ductal) breast carcinoma. Immunoreactivity specific for p53 was detected in the tumor cell nuclei. Perinuclear p53 immunolocalization is evident. Tumor cell nuclei were more frequently ball-like shaped or displayed an elliptic shape. Sequestered p53 localizes to large aggregates in the cytoplasm (avidin-biotin immunoperoxidase technique with mAb DO-7, counterstained with methyl green, ×320).

**Figure 3:**
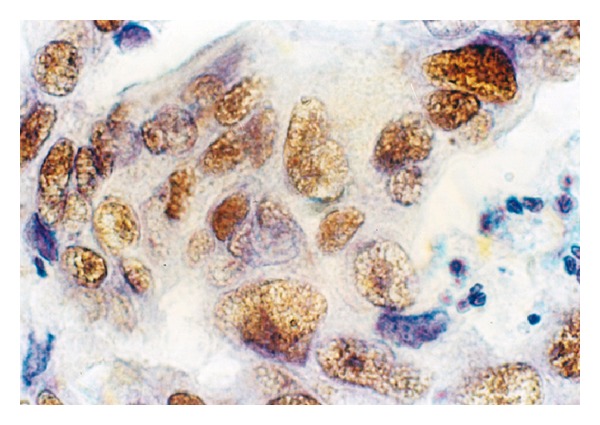
p53 immunoreactivity in infiltrating ductal carcinoma of the breast (invasive margins). Anti-p53 cell staining showing variation in the distribution and intensity of staining. In contrast to the darker, more sharply defined nuclear staining, a diffuse pattern of staining was identified in the cytoplasm of tumor cells. Larger and more irregular tumor cell nuclei had deeply invaginated surfaces (frozen section, immunoperoxidase with mAb DO-7, nuclear counterstain with haematoxylin, ×320).

**Figure 4:**
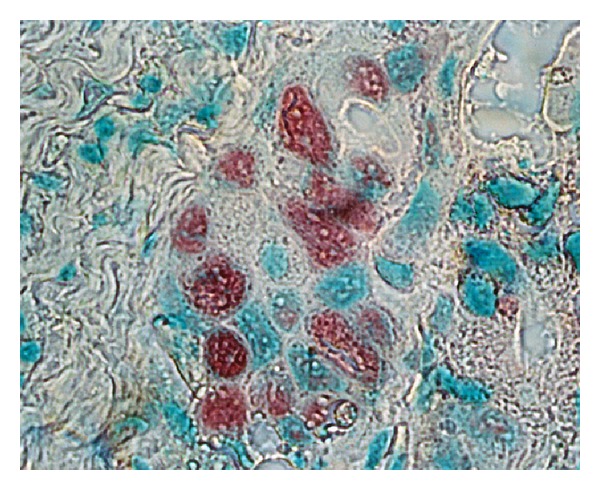
p53 immunostaining in malignant breast epithelial cells. Almost all neoplastic cell nuclei reveal strong accumulation of the p53 protein. p53-positive cells have red nuclei and a green staining underlines nuclei of unlabelled cells (frozen section, alkaline phosphatase antialkaline phosphatase—APAAP—technique, monoclonal antibody Pab240, fast red chromogen, counterstained with methyl green, ×250).

**Figure 5:**
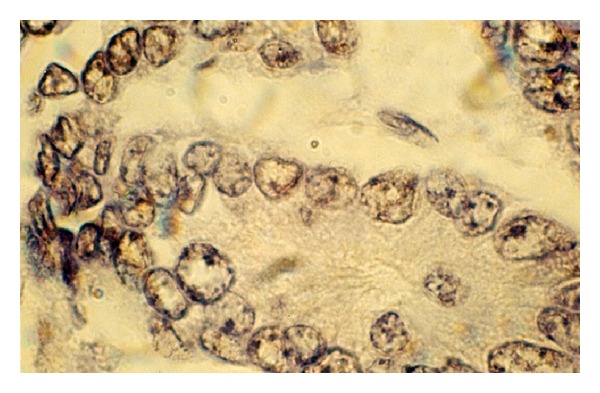
Unusual expression and cell compartmentalization of p53 protein in breast carcinoma cells. By immunohistochemical staining, p53 appears as discrete dot-shaped regions within the nucleus of the cell. p53 cancer-associated mutants localize to distinct domains in the nucleoli and at and around the nuclear membrane. The antibody reacted with a distinct perinuclear structure in almost all of the cells. Cytoplasmic p53 was observed as faint staining. Smaller tumor cell nuclei showed ball-like shape (frozen section, immunoperoxidase with antibody DO-7, nuclear counterstain with haematoxylin, ×250).

A distinct nuclear and cytoplasmic immunoreaction for p53 was judged as positive. Thirty of the 47 cases showed a positive immunoreaction with DO-7. Positive cells were distributed evenly in the cancerous tissue and there were 18 cases of 10–30% of positivity in tumor cells, 9 of 30–50%, and 3 of more than 50% in the 30 positive cases. In the positive cells the nuclear staining patterns were strong or diffuse (Figures 2 and 3). A similar localization is obtained with alkaline phosphatase-fast red detection (Figure 4). In contrast to the darker, more sharply defined nuclear staining, a diffuse pattern of staining was identified in the cytoplasm of tumor cells (Figure 3). Cytologic samples of nonmalignant lesions were always negative (Figure 1(a)). There was a strong association among nuclear p53 immunoreaction and histologic grade of breast cancer: 66% of grade 3 cases, 34% of grade 2 cases, and none (0%) of grade 1 cases. In the first series, the nuclear p53 immunoreaction was positive in three of four patients who died of breast cancer, whereas only one of 23 patients died among the negative cases. There was a significant difference in the overall survival between the group with a positive p53 immunoreaction detected by DO-7 and the other group with no p53 immunoreaction. In Serbian patients, we were able to confirm that nuclear p53 immunoreaction was more frequent in aggressive breast cancers, for example, those with histologic grade 3 and advanced clinical stage (Figure 1(b)). In fact, breast carcinomas that progress to the invasive stage tend to be more frequently p53-positive. It was also shown that a high pathological grade is statistically significantly associated with increased expression of p53 (*P* < 0.01, *t*-test). This may have diagnostic relevance because strong and diffuse p53 immunoreactivity has never been described in nonneoplastic breast tissues. Interestingly, we observed a greater nuclear staining for p53 protein at the invasive margins of the tumors. Tumor cell nuclei at the invasive front frequently displayed an elongated elliptical shape and had deeply invaginated surfaces (Figure 3). Smaller tumor cell nuclei showed ball-like shape (Figure 5). This result therefore is only one more indicator of the heterogeneity of malignant breast epithelial cells. The high resolution of the light microscopy provides a relatively precise detection of deep and narrow invaginations of nuclear membrane and also points to differences in the surface structure of the nuclei. Our data also suggested that immunohistochemistry for p53 protein would be clinically useful for predicting the prognosis of patients. Nuclear p53 immunoreaction was frequently detected in histologic grade 3 breast cancers, which have a poor prognosis. A high histologic grade of breast cancer is determined by the presence of structural and nuclear atypia and an increased number of mitotic figures in cancer cells. Therefore, alterations in p53 gene and overexpression of p53 protein are suggested to be involved in loss of differentiation, formation of nuclear polymorphism and a coarse chromatin pattern, and/or rapid growth of breast cancer.

One of the most intriguing findings is that mutant and/or wild-type p53 appear as discrete dot-shaped regions within the nucleus of breast carcinoma cells (Figure 5). In many malignant cells the nucleolar sequestration of p53 is evident (Figure 5).

**Figure 6:**
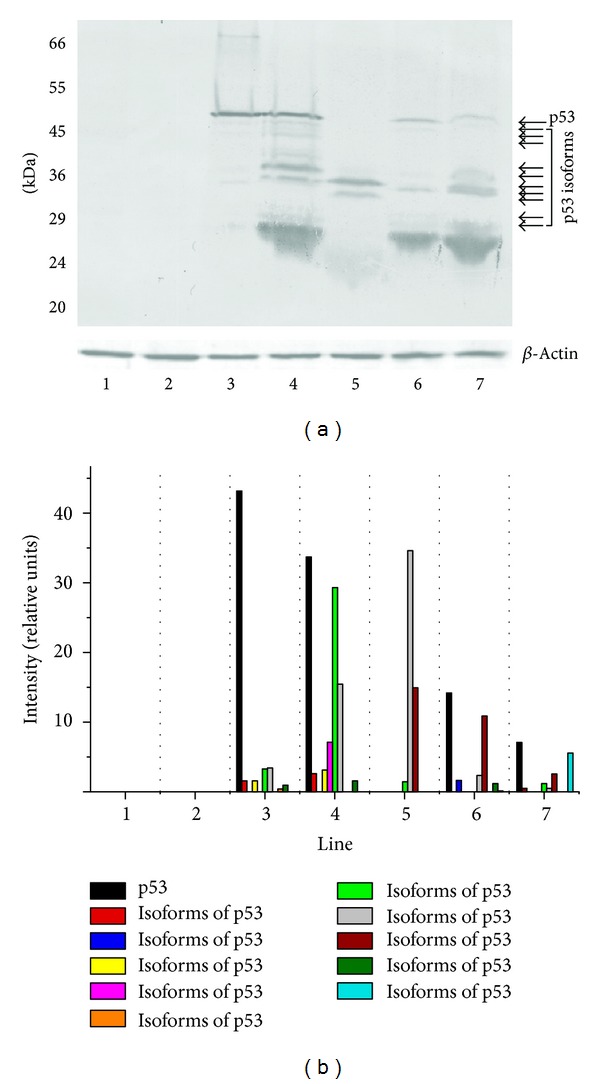
(a) Western blot analysis of p53 expressed in the nuclear fraction of human breast cancer cells revealed a wide spectrum of isoforms (49, 48, 47, 45, 36, 35, 34, 29, 28, and 24 kDa). These isoforms were not present in nontransformed cells (control cases, lines 1 and 2). Only the Δ133p53*β* (35 kDa) and ~36 kDa isoforms are present at higher levels in the invasive breast carcinomas (lines 4, 5, and 7) compared to noninvasive cases (lines 3 and 6). The analysis suggested coexpression of mutant forms of p53 and/or wild-type p53 with p53 isoforms indicated. Expression of multiple p53 isoforms varied between phenotypically distinct subset of cases. In normal breast tissues, expression of p53 is either very low or not measurable. Equal amounts of nuclear proteins (50 *μ*g/lane) from extracts of normal and breast cancer tissues were separated on 12% SDS-PAGE and transferred to nitrocellulose membrane. Incubation with CM-1 rabbit polyclonal antibody (diluted 1 : 1000) was followed by incubation with biotinylated swine anti-rabbit Ig serum (diluted 1 : 1000) and strept ABComplex-HRP conjugated as described in Section 2. HRP was detected with DAB as chromogen. Bottom panel shows actin loading control. All Western immunoblotting experiments were performed at least twice with similar results. Lines 1 and 2—normal breast tissue. Lines 4, 5, and 7—invasive ductal breast carcinomas. Lines 3 and 6—noninvasive breast carcinomas. Molecular mass markers are presented on the left (MW). The position of the p53 is indicated by the arrow. The arrows denote the position of the p53 isoforms. (b) Relative abundance of p53 and its isoforms in the nuclear fraction of breast cancer cells. The levels of p53 and its isoforms in each sample were quantified using transmission scanning and analyzed by Gel-Pro Analyzer 3.1. Significant increase of the p53 and its isoforms (35 and 36 kDa) is evident in nuclear fraction of breast cancer cells.

When the CM-1 antibody that had been generated against full-length p53 (recognized 1–393 aa, in wild-type and mutant p53 protein forms) was used in our study p53, p53*β* (48 kDa), ΔNp53 (47 kDa), and Δ133p53*β* (35 kDa) were detected in breast carcinomas (Figure 6). The authors found that multiple bands ranging from 24 to 53 kDa were detected in p53-expressing but not in p53-nonexpressing cells (Figure 6) supporting the notion that multiple p53 isoforms are expressed in breast cancer cells. Western blot analysis of the nuclear fraction of breast cancer cells revealed the Δ133p53*β* (35 kDa) isoform to be the dominant form in the invasive breast carcinomas (Figure 6, lines 4, 5, and 7) compared to non-invasive cases (lines 3 and 6). Importantly, none of the tumors had the same p53 isoform expression pattern. The isoforms expressed also varied between individual tumors, indicating potential roles of these p53 variants in human breast cancer. In fact, expression of multiple p53 isoforms varied between phenotypically distinct subset of cases. The presence of Δ133p53*β* (35 kDa) and ΔNp53 (47 kDa) isoforms is of critical importance in breast cancers with a wild-type p53.

We confirm the previously described variants in breast cancer tissues (p53*β*, ΔNp53, and Δ133p53*β*) and novel isoforms (49, 45, 36, 34, 29, 28, and 24 kDa), thereby, further enlarging combinatorial possibilities. Our experiments cannot determine whether the identified p53 isoforms associate with p53 individually or as part of a multicomponent complex. The protein level of each band was quantified by transmission scanning and analyzed by Gel-Pro Analyzer 3.1 (Figure 6(b)).

## 4. Discussion

In this study we used a panel of anti-p53 antibodies (DO-7, CM-1, and PAb240) for cell staining and found abnormalities in at least 30% of cases. DO-7 has shown to be one of the most sensitive for the detection of p53 overexpression [18]. Other literature data also support that rabbit p53 antibody CM-1 is highly sensitive for the p53 staining [11]. In addition, the CM-1 antibody is polyclonal and theoretically should recognize all forms of p53. It is widely accepted that, in normal tissues, p53 protein is present in such low quantities that it is not readily detectable by immunochemical techniques. Thus, the accumulation of p53 protein in normal breast epithelial cells is a highly unusual finding because the abnormally stabilized p53 protein has only been associated with malignant disease and DNA damage. Moreover, the half-life of p53 is extended from several minutes to several hours which is thought to be characteristic of a transformed phenotype.

The monoclonal PAb240 antibody reliably detects a wide variety of p53 mutations and these mutations have a common effect on the structure of p53. An antibody PAb240 does not immunoprecipitate wild-type p53.

Extensive evidence has shown that mutation or functional inactivation of the tumor suppressor p53 is an almost universal feature of human cancer. Previous studies suggest that mutant-type p53 has an influence on the prognosis of patients in many cancers including breast carcinoma and is associated with tumor staging, multidrug resistance, response to chemotherapy or radiotherapy, postsurgery recurrence, and metastasis [4, 6, 20]. Further analyses of point and truncated mutant expression products of the p53 gene have shown that mutant p53 proteins not only lose their tumor suppressive functions but may also gain new abilities that enhance tumorigenesis [21–25]. These data implicate transcriptional activation by mutant p53 as a key mechanism responsible for its oncogenic activity. Indeed, the p53 mutation is linked with chemoresistance and transformation to a more aggressive disease in many tumor types [26]. In the present study, we found that the higher expression levels of mutant p53 protein in most breast cancers analyzed were associated with more frequent lymph node metastasis, advanced TNM stage, and poor survival which is consistent with other reports [6, 27].

The role of *TP53* mutation as a prognostic marker is reviewed as well as its role as a predictor for therapy response. All data available on *TP53* mutation analyses of human breast carcinomas support an important role for *TP53* in mammary carcinogenesis [27, 28]. The use of different antibodies, staining standards, tumor material, scores for positivity, and the inclusion of variously selected groups of breast cancer patients might be the reason why the frequency of positive p53 staining ranges from 20 to 60% in the literature [29–31]. The rate of positive IHC was as high as 64% in our study and 36% cases had a complete lack of detectable staining. The nondetectable levels of p53 in the p53-negative tumors are either the very low wild-type levels or due to loss of p53 (deletion mutants) the overexpression of mdm2 and mdm2-mediated p53 degradation. This is in perfect agreement with the results described earlier [27]. Hence, excluding technical reasons, we tend to attribute the high rate of positive p53 staining in our study to the selection of patients with advanced stages of breast cancer (T3 and T4 tumors). We also hypothesized that expression profiling of p53 in breast cancer would lead to the identification of a molecular signature that can predict metastatic potential. Generally, the nuclear p53 immunoreaction was considered to reflect nuclear accumulation of mutant p53 protein which is coded by the mutated form of the p53 gene, and has a prolonged half-life. From these findings and our present ones (Figures 2, 3, and 4), it is suggested that breast cancer showing a positive nuclear p53 immunoreaction carries the mutated p53 gene and that mutation of p53 may play an important role as a mechanism for aggressive biological behavior of human breast cancer. Since p53 mutations could reflect a higher rate of proliferation and more advanced state of progression [24], breast tumors with these p53 alterations, as evidenced by nuclear accumulation of protein, could have a greater probability of having micrometastasis and thus a greater probability of recurrence [27]. Olivier et al. [27] hypothesize that p53 mutations (mp p53) are associated with decreased expression of thrombospondin 1 (TSP-1) and that decreased TSP-1 expression is associated with lymph node metastases. Subsequent analyses of large series of individual tumor types have, however, revealed a much more complex story [32–35].

The possibility that p53 status influences biological behavior was raised in an early study in which the presence of p53 mutations in aggressive breast cancer was demonstrated [36] and the majority of studies support the existence of an association between worse survival and the presence of p53 mutations. This association was confirmed in a comprehensive meta-analysis of the effect of somatic p53 mutations on prognosis in breast cancer [37]. However, the difficulty to link p53 mutation status to clinical outcome and cancer treatment can be explained by the fact that mutations of the p53 gene do not necessarily result in inactivation of p53 transcriptional activity [20]. Hence, 60% of the mutations that can occur in the p53 gene do not alter p53 transcriptional activity. Only 15% of the mutations lead to mutant p53 completely inactive in transactivation. In the remaining 25% mutants, p53 presents differential transcriptional activity. They can transactivate some promoters but are completely inactive on others. It is therefore important to establish whether p53 mutation is correlated with loss of p53 function to determine accurately p53 status in clinical studies.

A large number of studies have assessed the prognostic and predictive role of TP53 alterations in breast cancer yielding conflicting results. Two different methodologies have been used to assess TP53 alterations: DNA sequencing and immunohistochemistry (IHC). Most *TP53* alteration found in breast carcinomas are point mutations leading to the synthesis of a stable, malfunctional, and nondegradable protein that accumulates in tumor cells and thus can be detected by IHC. The correlation between p53 protein accumulation measured by IHC and *TP53* mutation detected by sequencing is, however, less than 75% in breast carcinomas [38]. The reason for this is that not all mutations yield a stable protein and some mutations result in protein truncation and are thus not detected by IHC. Studies that have used sequencing to detect mutations all showed a strong association to survival, whereas most studies using IHC failed to detect such an association [39]. Careful studies of microdissected tumor material have shown that TP53 mutations can occur in ductal carcinoma *in situ* (DCIS) before the development of invasive breast cancer, and that the frequency increases from around 0% in low-grade DCIS to 30–40% in high-grade DCIS [40–42]. These results point to an important role of TP53 alterations early in the carcinogenic process of the breast. The frequency of TP53 mutations reported in breast tumors ranges from 15 to 71% [43]. Significant differences are seen among populations, although the different distribution of various stages, as well as whether the whole gene was screened or only the conserved region from exons 5–8, may have influenced the frequency found in the various studies. The frequencies in node-negative patients are on the whole considerably lower (15–18%) than in node-positive patients and large tumors from patients with advanced disease have a higher frequency of mutations than small tumors [44]. An increased frequency during tumor progression has also been observed [4, 45]. Analysis of the p53 mutation spectrum [46, 47] indicates that ~5–15% of the p53 mutations occur in the C-terminal domain (CTD) during human tumorigenesis. Most of the somatic mutations in the p53 CTD are either nonsense mutations or lead to frameshifts, resulting in a truncated protein. The results have revealed that all mutants have impaired apoptotic activity when compared with wild-type p53 [48]. These and other data indicate that CTD of p53 is an important component of p53-mediated apoptosis and cell growth arrest and that inactivation of the apoptotic function, but not the inhibition of growth, is an important step during human tumorigenesis [48].

In many malignant cells, the nucleolar sequestration of p53 is evident (Figure 5). These observations suggest a link between p53 breast cancer-associated mutants and ribosomal biogenesis. The nucleolus is the major site of ribosome biogenesis and we now show that mutant and/or wild-type forms of p53 associate with nucleolar structures (Figure 5). The subpopulation of nucleolar p53 may be involved in ribosomal processing. Nucleolar transcription is considered to occur in the transition zone between the fibrillar centres and the dense fibrillar component, whilst rRNA processing progresses from the dense fibrillar component to the granular component [49]. Our results are in agreement with a similar observation reported in the literature that high intensity fibrillarin spots reveal the dense fibrillar component [49]. Interestingly, we observed nucleolar p53 spots both in high and low fibrillarin regions (Figure 5), thus supporting the idea that mutant and/or wild-type forms of p53 might be associated with several nucleolar functions. It is likely that the answer lies within the realm of the nucleolus, which is the central factory for ribosome biosynthesis and assembly and where the key regulators of p53 (Arf, mdm2, L5/L11/L23, and p53 itself) reside at some point [50, 51]. p53 mutants specifically bind to nuclear matrix attachment region (MAR) DNA elements. MAR elements constitute important higher-order regulatory elements of chromatin structure and function. By binding to these elements, mutant forms of p53 could modulate important cellular processes, like gene expression, replication, and recombination, resulting in phenotypic alterations of the tumor cells [52–55].

Although the interpatient variability was large, we found that tumor cell nuclei at the invasive front frequently displayed an elliptic shape and had deeply invaginated surfaces (Figure 3). In contrast, the majority of the tumor cell nuclei analyzed were ball-shaped (Figures 2 and 5). It is therefore possible that the nuclear surface and area are reliable markers for terminal stages of the biochemical differentiation of breast carcinoma. It is noteworthy that regulation of cell differentiation is most often impaired in malignant tumors and may represent a key mechanism for the progression of the disease. It should be remembered, however, that the disruption of normal epithelial differentiation represents a primary event in the initiation of a malignant phenotype. Indeed, a switch in differentiation state can play a major causal role in tumor evolution by altering the selection pressure for p53 mutation [56].

In keeping with its function as a transcription factor, p53 is predominately a nuclear protein. However, a fraction of p53 can be found in the cytoplasm, suggesting a transcriptionally independent function for this pool of p53. The binding of the various forms of p53 to plasma membranes (Figures 3 and 5) suggests that a subset of membrane proteins and a variety of molecules including phospholipids and proteoglycans have binding sites for these structurally distinct forms of p53. Membrane lipid domains have been proposed to be involved in a variety of different functions including signal transduction, lipid transport and metabolism, and cell growth. Thus, the various effects of p53 binding to these membrane molecules suggest other roles of p53 than at the transcriptome level. Binding of p53 to the membrane could also have consequence on p53 itself. p53 signaling, therefore, appears “soft-wired”, modular, and highly flexible with substantial overlap between different response pathways. The cellular background likely profoundly affects the nature of the response.

To investigate whether expression of various p53 isoforms might be responsible for clinical observations, thirty randomly selected breast tumors were analyzed for expression profiles of different p53 isoforms, by Western blot analysis (Figure 5). Whereas normal human breast does not express any of the corresponding isoforms, different p53 isoforms are detected in the majority of breast tumors at apparently different levels (Figure 5). This is most probably true and such hypothesis is consistent with experimental evidence showing abnormal expression of p53 isoforms in breast tumors [57]: p53*β*, which lacks the carboxy-terminal oligomerization domain due to alternative mRNA splicing; Δ133p53, which lacks the amino-terminal transactivation and proline-rich domains due to the transcription from an alternative promoter in intron 4; Δ40p53, also named p47 or ΔNp53, as an amino-terminally truncated p53 isoform deleted from the first 40 amino acids. ΔNp53 protein can be generated either by an alternative splicing of the intron 2 or by alternative initiation of translation [57] and can act in a dominant-negative manner towards wild-type p53 inhibiting both p53 transcriptional activity and p53-mediated apoptosis [58]. Therefore, the human p53 gene can encode at least nine different p53 protein isoforms [57]. ΔNp53 contains a shorter N-terminus (lacking 39 aa) and Δ133p53 lacks the whole N-terminal region plus part of the DNA-binding domain [59].

Rohaly et al. [60] attempt to demonstrate endogenous Δp53 expression using a panel of p53 monoclonal antibodies. The authors could detect a p53 band at 45 kDa with DO-1, which was interpreted as proving the expression of the Δp53 isoform. However, this should be interpreted with caution, as Bourdon et al. [57] showed that p53*β*, p53*γ*, and Δ40p53 migrate all at 45 kDa. Therefore, the 45 kDa band identified by Rohaly et al. [60] is probably composed of a mix of Δp53, p53*β*, p53*γ*, and Δ40p53. Δp53, which lacks a conserved domain of p53 in the DNA-binding domain, was reported to be transcriptionally active towards some p53 target genes and to be critical for the intra-S phase checkpoint [60]. Contrary to previous publications, Wan and Poon [61] reported with strong experimental evidences that Δp53 isoform lacks intrinsic transcriptional activity and lacks dominant-negative activity towards full-length p53. This is probably because Δp53 is not imported into the nucleus and stays in the cytoplasm. Therefore, further studies will be required to establish expression of the endogenous Δp53 isoforms and its biological relevance. By using the CM-1 antibody (which detects p53, p53*β*, and Δ133p53), Fujita et al. [62] showed that p53*β* and Δ133p53 were expressed less abundantly than full-length p53 but were still at readily detectable levels in human fibroblast strains MRC-5 and WI-38. Bourdon et al. [63] have reported an analysis of expression of p53*β* and p53*γ* in relation to clinical and pathological markers and disease outcome in a cohort of 127 randomly selected primary breast tumors. p53*β* was associated with tumor estrogen receptor (ER) expression, and p53*γ* was associated with mutation of the p53 gene. The patients group with the mutant p53 breast tumor-expressing p53*γ* isoform had low cancer recurrence and an overall survival as good as that of patients with wild-type p53 breast cancer Conversely, patients expressing only mutant p53, without p53*γ* isoform expression, had a particularly poor prognosis.

When the CM-1 antibody, that had been generated against full-length p53, was used in our study, p53, p53*β* (48 kDa), ΔNp53 (47 kDa), and Δ133p53 (35 kDa) were detected in breast carcinomas (Figure 6). This analysis demonstrated that the CM-1 antibody is specific for the unique epitope at the ΔNp53 and *β* isoform subclasses, p53*β* and Δ133p53*β*. It was published that the 133p53 isoform is a direct p53 target gene that modulates p53 tumour suppressor activity [57]. Since the majority of the tumors analyzed had an abnormal pattern of p53 isoform compared to that of normal breast tissue, the authors speculate that an imbalance in isoform expression may thwart p53's tumor suppressive capabilities, thereby accelerating the tumorigenic process in the absence of p53 mutation. It is noteworthy that the dominant-negative effect of N-terminally truncated isoforms on the activity of full-length isoforms has been demonstrated [57]. Indeed, Bourdon et al. [57] found that tumors that retain wild-type p53 often express an abnormal balance of p53 isoforms. Since N-terminally truncated p53 isoforms can regulate p53 activity, an alteration in the ratio of different p53 isoforms may predispose to cancer and might even explain the lack of correlation between p53 mutational status and response to anticancer therapies. Whereas only 25% of breast tumors express mutant p53, p53*β*, and p53*γ*, expressions are frequently lost (60%) and Δ133p53 is frequently overexpressed in breast tumors [57].

The most parsimonial explanation of the results described above is that p53 mutation (or the associated elevation of cellular p53 protein levels) is the commonest genetic alteration detected in primary breast carcinoma cells. Overexpression of p53 in primary breast cancer is associated with high tumor grade and nodal metastases. It should be pointed out that there is also an increase in the level of p53 isoforms in the majority of the invasive breast carcinomas studied in detail. It is possible that the dominant-negative ΔN forms of p53 were generally detected in these tumor cells so a role in the regulation of p53 function has been hypothesized. The discovery that ΔNp53 is the dominant isotype in breast carcinoma does suggest a plausible mechanism by which the p53 present in these tumors is prevented from functioning as a transactivator. If the two proteins, dominant-negative variants that lack the amino-terminal transactivation domain, compete for binding sites on DNA, ΔNp53 and Δ133p53*β* will very effectively act as a dominant-negative repressor of p53 function. Based on our experience, structured and funded qualitative research is required to establish why such high levels of p53 isoforms exist in invasive breast carcinoma cells. These cells should also serve in clarification of the process leading to transformation by mutant forms of p53. The most straightforward conclusion is that many questions remain to be answered about the tumor derived p53 mutants and the intricate network involving members of the p53 family. Further work in this area should help provide a new insight into the mode of action of mutant forms of p53 and p53 isoforms in breast carcinoma cells as well as their potential clinical relevance.
